# New Considerations in the Use of Collagenase Clostridium Histolyticum for the Treatment of Cellulite

**DOI:** 10.1093/asjof/ojad068

**Published:** 2023-07-18

**Authors:** Jeremy A Grekin, Joel L Cohen, Michael S Kaminer

We appreciate the thorough review of collagenase clostridium histolyticum (CCH) and its use in clinical practice for the treatment of cellulite by Graivier et al.^1^ The authors highlight the need for alternative injection techniques in the treatment of the thighs (when compared with the buttocks) without commenting on the anatomic reasons why this may be necessary.^1^ We would like to emphasize the importance of such alternative injection techniques by presenting an interesting adverse event currently unreported in the literature.

A 58-year-old female patient presented to the clinic for the evaluation of cellulite after completing treatment with CCH by an outside, nonphysician provider. The patient reported completing 3 rounds of treatment for areas of concern along the buttocks as well as both the posterior and anterior thighs. She was disappointed with the results and complained of bruising as well as persistent pain along bilateral anterior thighs with ambulation, as well as typical levels of exercise. Following thorough evaluation and imaging, it was discovered that the patient had developed a moderate 1.0 cm tear of the right and a moderate-to-high grade 1.5 cm tear of the left rectus femoris muscles. The patient denied any significant trauma or other injury that might have triggered the bilateral tear, making treatment of the anterior thighs with CCH the most likely culprit.

Of note, CCH carries an additional FDA indication in the United States for the treatment of other collagen-associated disorders, including Dupuytren's contracture and Peyronie's disease, where there are reports of severe adverse events including complete tendon rupture and corporeal or penile fractures, respectively.^2,3^ We could find no translatable reports of skeletal muscle rupture in the literature with the use of CCH for cellulite. It should be emphasized, though, that CCH currently carries approval for the treatment of cellulite in the buttocks.^4^

Types I and III collagen, the target of CCH, are prominent components of skeletal muscle endomysium and perimysium, which could make damage to skeletal muscle possible with exposure to sufficient doses of CCH.^4^ Anatomically, skeletal muscle may lie much more superficially along the thighs compared to the buttocks; hence, injection in these areas should be performed much more superficially to minimize the risk of direct intramuscular injection, particularly anteriorly. The rectus femoris is the most superficial component of the quadriceps muscle and thus may be particularly vulnerable to CCH injection and subsequent rupture, as observed here.^5^

With continued investigation and use of CCH for the treatment of cellulite in broader anatomic locations, it is likely that the overall risk of skeletal muscle complications will become more evident. It is also probable that a more detailed evaluation of the underlying tissues should be utilized when administering CCH to more anatomically sensitive areas, such as the anterior thighs. Doing so will further guide and instruct users on alternative injection techniques such as injecting much more superficially, which may be necessary to avoid complications, such as muscle tears as observed in this case. Given the relatively new indication and use of CCH for the treatment of cellulite, it is important for providers to continue to report on all possible adverse events and how best to mitigate them.
